# P-2367. Qualitative Analysis of Barriers to Outpatient Antiviral Treatment for COVID-19 and Influenza Patients Observed by Infectious Disease Specialists in North America, 2024

**DOI:** 10.1093/ofid/ofae631.2517

**Published:** 2025-01-29

**Authors:** Souci Louis, Dennis Wang, Jordan Singleton, Dallas J Smith, Anastasia S Lambrou, Susan E Beekmann, Philip M Polgreen, Shikha Garg, Jessica Ricaldi, Timothy M Uyeki, Scott S Santibanez, Pragna Patel

**Affiliations:** CDC - Atlanta, GA, Atlanta, Georgia; CDC, Atlanta, Georgia; CDC, Atlanta, Georgia; Mycotic Diseases Branch, Centers for Disease Control and Prevention, Atlanta, Georgia; CDC, Atlanta, Georgia; University of Iowa, IOWA CITY, Iowa; University of Iowa Carver College of Medicine, Iowa City, IA; Centers for Disease Control and Prevention, Atlanta, Georgia; CDC - Atlanta, GA, Atlanta, Georgia; Centers for Disease Control and Prevention, Atlanta, Georgia; CDC, Atlanta, Georgia; CDC, Atlanta, Georgia

## Abstract

**Background:**

Antiviral medications for COVID-19 and influenza can mitigate disease severity in high-risk outpatients if taken early in the course of illness yet are underutilized. We sought to understand barriers to providers prescribing these medications.

Table

**Figure:**
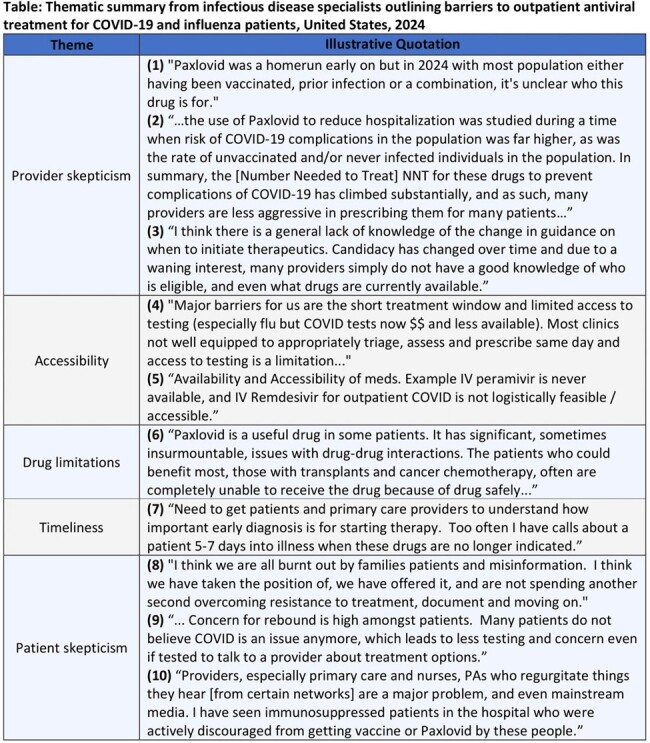

Thematic summary from infectious disease specialists outlining barriers to outpatient antiviral treatment for COVID-19 and influenza patients, United States, 2024

**Methods:**

We conducted an on-line survey regarding knowledge, attitudes, and practices of prescribing antiviral treatment for outpatients with COVID-19 and influenza. Respondents were asked about perceived barriers among providers treating COVID-19 and influenza outpatients in their institutions. Questions were structured using a Likert scale and were analyzed using a thematic analysis approach in Microsoft Excel.

**Results:**

Of 1,898 infectious disease specialists across the United States and Canada who received the survey between 1/10/24 to 2/5/24, 565 (30%) responded, of whom 93% were infectious disease physicians and 7% were healthcare professionals. Surveyed physicians worked in university (45%), non-university teaching (24%), community (20%), city/county (4%), outpatient (0.4%), and veteran affairs hospital settings (6%) and 144 (25%) provided free text responses. The primary barrier to prescribing antivirals was provider skepticism (47%), due to patient symptoms deemed too mild for treatment and needing more evidence about effectiveness. This was followed by perceptions of limited accessibility (31%) related to high cost, limited hospital access, and difficulty administering some medications. Pharmacologic limitations (18%) were concerns about drug interactions, side effects, and incomplete medical history. Other barriers were timeliness of treatment within a short therapeutic window (15%) and patient skepticism (13%). Additional free text responses described successful hospital protocols and suggestions for encouraging antiviral prescribing.

**Conclusion:**

These themes demonstrate a need for better education of providers about antiviral risks and benefits as well as improved access and coverage of life-saving medications. Infectious disease specialists provided useful insights which can help to shape clinical recommendations, future research for antiviral therapeutics, and public health messaging for improved patient care.

**Disclosures:**

Philip M. Polgreen, MD, Eli Lily: Advisor/Consultant|Pfizer: Grant/Research Support

